# Development of a Measure of Informal Workplace Social Interactions

**DOI:** 10.3389/fpsyg.2019.02043

**Published:** 2019-09-20

**Authors:** Carolyn J. Winslow, Isaac E. Sabat, Amanda J. Anderson, Seth A. Kaplan, Sarah J. Miller

**Affiliations:** ^1^School of Public Health, University of California, Berkeley, Berkeley, CA, United States; ^2^Department of Psychological and Brain Sciences, Texas A&M University, College Station, TX, United States; ^3^Fors Marsh Group, Arlington, VA, United States; ^4^Department of Psychology, George Mason University, Fairfax, VA, United States

**Keywords:** social interaction, scale development, measurement, organization, workplace

## Abstract

A measure of informal, non-task-related workplace social interactions that captures both the frequency of interactions and the positive affect that can accompany such interactions was developed and validated. In two samples of employees (*N* = 188 and *N* = 315, respectively), the factor structure, reliability, and incremental predictive validity of the newly developed measure were evaluated. Results support the anticipated two-factor structure, demonstrate strong psychometric properties, and reveal that the new measure explains additional variance in employee outcomes (job satisfaction and job-related positive affect). This newly developed, 16-item scale provides a psychometrically sound measure for researchers and organizations to use in assessing, and potentially improving, two dimensions of workplace social interactions.

The desire to feel connected to others has long been identified by psychologists as a basic human need (e.g., Maslow, 1943; Baumeister and Leary, 1995; Sheldon and Gunz, 2009). Within the workplace, social relationships are important for various attitudinal, well-being, and performance-related outcomes (Chiaburu and Harrison, 2008; Basford and Offermann, 2012). Existing measures of workplace social relations, such as measures of workplace friendship (Nielsen et al., 2000) and other forms of peer social support (Haynes et al., 1999), assess social connections associated with instrumental outcomes. However, evidence from outside the organizational domain also demonstrates the benefits of the mere occurrence of interpersonal interactions. Here, we translate this research into the organizational arena, suggesting that informal workplace social interactions represent affective events. It is largely through the accumulation of these events that the salubrious outcomes of more enduring workplace relationship factors manifest. Specifically, this brief report offers a theoretical account – and corresponding measure – of informal, non-instrumental workplace social interactions.

## Conceptualizing Informal Workplace Social Interactions

We propose a measure of informal, non-instrumental workplace social interactions, defined as the extent of one’s participation in casual, non-task/work-related social interactions in the organization. Additionally, the proposed measure assesses the emotions experienced with and/or subsequent to these interactions. Notably, unlike existing measures of workplace social relations, the present measure seeks to capture engagement in – and affective reactions to – interpersonal exchanges among coworkers that do not necessarily help workers to cope with the demands of work or serve other goal-oriented purposes.

The behavioral dimension of the measure assesses interactions in the form of job-unrelated casual contact and conversations. This type of social contact has been recognized by scholars as one of the “latent functions” (p. 25) of work that serves to promote employee well-being (Jahoda, 1982). Such informal social interactions with others may promote well-being by cueing a sense of belonging and also by boosting the moods of those engaging in the encounter. Indeed, much empirical research suggests a connection between mere social contact/activity and positive emotional states (McIntyre et al., 1991; Lucas, 2001; Sandstrom and Dunn, 2014; Park and Hinsz, 2015), as well as subjective well-being more generally (Okun et al., 1984; Appau et al., 2019). Thus, this research suggests that social interactions that do not necessarily entail the provision of support (emotional, informational, or otherwise) can also serve as an important antecedent to employee well-being and associated outcomes.

Beyond assessing informal interactions themselves, we also assessed *affect* associated with such interactions. According to Affective Events Theory (AET; Weiss and Cropanzano, 1996), affective work events serve as proximal causes of affective reactions that influence distal job attitudes. Although the theory is somewhat ecumenical in defining work events, discrete informal social encounters may readily be considered one type of affective event. Thus, we suggest that, consistent with the abovementioned research reporting the mood-enhancing benefits of minimal interactions (e.g., Lin and Kwantes, 2015), it is critical to assess the emotional states resultant from casual workplace social interactions. Assessing affect in conjunction with behaviors is also important because some individuals (e.g., those who are more extraverted or agreeable) may differ in their affective reactions to the same interactions (Larsen and Ketelaar, 1991; Letzring and Adamcik, 2015).

## Comparison to Existing Measures

Several constructs and measures are conceptually related to the proposed measure of informal workplace social interactions. These constructs include: social connectedness (Lee and Robbins, 1995), workplace friendship (Methot et al., 2016), and social support from peers in the workplace (Chiaburu and Harrison, 2008).

First, we distinguish the proposed measure from Lee and Robbins’ (1995) measure of social connectedness, which is part of a broader measure of belongingness. Items in the social connectedness subscale reflect social embeddedness, the degree to which one perceives a general emotional distance between self and others. For example, the measure contains items such as, “I catch myself losing all sense of connectedness with society,” and “I feel so distant from people” (p. 236). Thus, in contrast to the proposed measure, the Lee and Robbins’ (1995) measure does not reflect one’s engagement in, or affective reactions to, specific interpersonal exchanges, and instead assesses a sense of belonging to society and others more generally.

The newly developed measure is also conceptually distinct from measures of workplace friendship and peer social support. Unlike the Lee and Robbins’ measure – but similar to the proposed measure – measures of peer social support and workplace friendship do assess, to some degree, interactions with individuals in one’s immediate social environment. However, as noted earlier, both workplace social support and friendship measures capture established, trusting relationships that are accessed and maintained to meet work-related emotional and task-related challenges/needs. Consistent with this interpretation, social support is commonly conceptualized as “a social network’s provision of psychological and material resources intended to benefit an individual’s ability to cope with stress” (Cohen, 2004, p. 676). Similarly, workplace friendship is understood as a “multiplex” or multifaceted relationship where as “a personal, affective relationship coincides with a business relationship” (Methot et al., 2016, p. 312). Taken together, we suggest that both workplace social support and friendship contrast with the proposed measure, which aims to capture “pure” social contact/interactions that lack the support functions of workplace friendships and peer social support. This type of non-supportive contact includes: talking to colleagues about shared interests, making jokes, and discussing non-work leisure activities. Although these informal social interactions may indirectly provide support (by serving, for example, as distractions), we suggest that these types of informal interactions lack the explicit emotional intentions of friendship and socially supportive interactions more generally. Taken together, the current measure aims to assess informal, non-task workplace social interactions and the emotional experiences associated with those interactions, whereas existing measures assess either a general sense of belonging (i.e., the Lee and Robbins (1995) measure of social connectedness) or more instrumental, socially supportive workplace relationships (i.e., measures of workplace friendship and social support).

## Method

### Item Development

We used a deductive approach for developing items, allowing specific items to be generated from our theoretical definition. After the initial items were written, they were subjected to pretesting and cognitive interviewing to evaluate content validity and item clarity. We followed Hinkin (1998, p. 109) procedures to gauge the adequacy of our items. Specifically, five psychology graduate students assisted with pretesting by independently sorting items according to whether they reflected the proposed behavioral dimension, the proposed affective dimension, or the Lee and Robbins’ (1995) definition of social connectedness (given this measure’s predominance in the literature). Results revealed that most items were appropriately categorized except for two affective items, which were subsequently removed.

Following pretesting, cognitive interviewing was conducted with a separate group of employed adults (Caspar et al., 1999). The majority of the items were perceived as intended except for seven behavioral and six affective items, which were subsequently removed. This revision process resulted in a list of 23 items (11 behavioral and 12 affective items).

### Participants and Procedures

The study was approved by George Mason University’s Institutional Review Board, and we obtained informed written consent from all participants, who were recruited from Amazon’s Mechanical Turk (MTurk). Eligibility requirements included being at least 18 years old and working 20 hours more per week for at least three months in an organization that provided opportunities for social interactions. A total of 614 participants completed the 15-min survey.

### Measures

Several measures, including the key measures of belongingness, workplace social support, and workplace friendships as defined earlier, were investigated for evidence of convergent, discriminant, and predictive validity. Participants were instructed to respond to all measures (except for personality and the control variables) regarding their experiences/emotions at work over the past 3 months to allow for sufficient occurrence of representative social interactions. All responses were made on a five-point Likert scale.

### Convergent and Discriminant Validity

#### Social Connectedness

We included the eight-item social connectedness scale of Lee and Robbins’ (1995) measure of Belongingness. We expected the proposed measure to demonstrate a moderate, positive relationship with this measure, but that the two would not be wholly redundant because, as explained previously, the Lee and Robbins’ (1995) measure reflects a sense of belonging to society more generally.

#### Workplace Friendship

The 12-item Workplace Friendship Scale (Nielsen et al., 2000) was used to assess both opportunities for and prevalence of friendships formed at work. We expected that the newly developed scale would show a strong positive relationship with this scale, particularly because the items in the friendship prevalence dimension of the scale (which contains items such as “I can confide in people at work” and “I feel I can trust my coworkers a great deal”) may closely relate to those in the affective dimension of the proposed measure. However, we expected some uniqueness, especially for the proposed behavioral dimension.

#### Workplace Social Support

Support was measured via a four-item measure developed by Haynes et al. (1999) that asks respondents to what extent they can “count on colleagues” for activities such as “talking about my problems at work” and “helping with a difficult task.” We expected a moderate positive correlation between this and our measure.

#### Workplace Isolation

Workplace isolation was assessed using the six-item Colleague subscale developed by Marshall et al. (2007). This measure contains items such as, “I had people around me at work.” We expected a moderate negative correlation between this and the new measure because employees should perceive less isolation from coworkers and the organization the more they engage in workplace interactions.

#### Extraversion

Extraversion was assessed using the relevant 10 items from the 50-item International Personality Item Pool (Goldberg, 1999). A sample item is, “I feel comfortable around people.” This measure was included to determine whether engagement in informal social interactions at work varies as a function of extraversion, with more extraverted employees engaging in more informal social interactions. We expected a moderate positive correlation between this and the new measure.

### Predictive Validity

According to AET, affective work events lead to affective reactions that influence more distal job attitudes. Based on this theory, we examined how our measure of informal social interactions predicts the following attitudinal and well-being-related measures: job-related affective well-being, happiness, and job satisfaction. Positive and negative affective well-being was assessed using a 12-item version of the Job-Related Affective Well-Being Scale (Van Katwyk et al., 2000), which assesses the frequency of emotional reactions (e.g., “excited,” “discouraged”) to one’s job. Subjective happiness was measured using the four-item Subjective Happiness Scale (Lyubomirsky and Lepper, 1999; e.g., “Some people are generally very happy…to what extent does this characterization describe you?”). Finally, job satisfaction was measured using Brayfield and Rothe’s (1951) five-item measure of overall job satisfaction, containing items such as, “I found real enjoyment in my work.” We expected both dimensions to predict these outcomes.

### Control Variables

We included two items measuring positional tenure and telework frequency to serve as control variables for predictive validity. Tenure was controlled for because employees’ level of social integration and interaction could be a function of time spent in the organization and engaging with coworkers. Additionally, we controlled for the amount of time spent working from home to remove potential variance in outcomes due to differential opportunities for workplace social interactions among teleworkers (Cooper and Kurland, 2002).

## Results

Data from 614 participants residing in the U.S. were collected. Fifty-six participants did not meet the eligibility criteria and were removed from the data, along with 18 participants with a significant amount of missing data and five participants who took more than 1 h to complete the survey. Moreover, consistent with best practices for safeguarding against inattentive responding among MTurk participants (Fleischer et al., 2015), 32 participants who failed two attention checks (e.g., “please select ‘strongly agree’ if you are paying attention”) were removed. In sum, 111 of the initial 614 participants were removed, for a total of 503 eligible participants. The average age of participants was 33.78 (*SD* = 11.25). Forty-five percent identified as female, 54.5% identified as male, and 0.5% did not report this information.

### Descriptive Statistics and Item Reduction

Descriptive statistics were computed for all items (see Table 1, which also contains the initial and final list of items). Item means ranged from 3.00 to 4.00, with standard deviations approximately equal to 1. Item distributions were normal, with a few items showing a slight negative skew (e.g., 1 and 23).^1^

**Table 1:** Item descriptive statistics factor loadings, and communalites.

Item	Mean	SD	Behavioral dimension	Affective dimension	Communality
1. I talked to people in my organization about shared interests.	3.67	0.81	0.70	0.56	0.650
2. I made jokes on the job with people in my organization.	3.60	0.93	0.72	0.35	0.610
3. I often talked with people in my organization about topics that are not related to work.	3.54	0.92	0.77	0.51	0.748
4. I connected with people in my organization by sharing personal information and experiences.	3.42	0.95	0.78	0.39	0.597
5. I discussed the events of my non-work time (i.e., weekend and/or evening) with people in my organization.	3.46	0.92	0.82	0.41	0.722
6. I discussed the events of my time off from work (e.g., holidays and/or vacations) with people in my organization.	3.42	0.89	0.78	0.39	0.706
7. I engaged in social interactions (i.e., talking in person or over the phone, messaging, emailing, or communicating through other social media) with people in my organization throughout the work day.^*^	3.78	1.00	—	—	—
8. I participated in social activities with people in my organization during the work day (e.g., singing happy birthday to a coworker, going to the gym, eating lunch with others).^*^	3.31	1.06	—	—	—
9. I engaged in social interactions with people in my organization outside of the work day (i.e., talking in person or over the phone, messaging, emailing, or communicating through other social media).^*^	2.90	1.14	—	—	—
10. I participated in social activities with people in my organization outside of the work day (e.g., happy hours, movies, shows).^*^	2.63	1.20	—	—	—
11. I maintained friendships with other people in my organization.^*^	3.49	0.97	—	—	—
12. I enjoyed interacting with people in my organization.	3.88	0.85	0.39	0.84	0.791
13. I gained a sense of happiness when interacting with people in my organization.	3.75	0.91	0.44	0.84	0.726
14. My mood improved when I interacted with people in my organization.	3.66	0.97	0.40	0.80	0.654
15. Talking with people in my organization during work made me feel happy.	3.77	0.95	0.49	0.89	0.818
16. Being a part of social groups at work improved my mood.	3.74	0.94	0.54	0.81	0.677
17. I felt content when I was around people in my organization.	3.72	0.85	0.36	0.71	0.520
18. I felt an improvement in my mood when I connected with people in my organization.	3.65	0.91	0.46	0.77	0.611
19. I enjoyed learning about the interests of people in my organization.	3.76	0.90	0.38	0.78	0.640
20. I enjoyed making friends with the people in my organization.^*^	3.77	0.89	—	—	—
21. I felt good when people in my organization talked to me at work.	3.91	0.82	0.42	0.81	0.668
22. I was happy when other people in my organization included me in social interactions at work.	3.88	0.86	0.55	0.78	0.723
23. I liked talking to people in my organization about my personal experiences.^*^	3.71	0.98	—	—	—

*For the behavioral dimension, response options ranged from 1 (*Never*) to 5 (*Very frequently*). For the affective dimension, response options ranged from 1 (*Strongly disagree*) to 5 (*Strongly agree*). SD, standard deviation*.

* *Item was tested but not retained in final scale*.

### Exploratory Factor Analyses

After examining the descriptive statistics for the items in the measure, we conducted a series of EFAs with a randomly selected sample of 188 respondents^2^. (The remaining 315 respondents were used to conduct CFAs.) All 23 original items were used to conduct an EFA with Principal Axis Factoring and oblique (Promax) rotation given expectations that the behavioral and affective dimensions would be correlated. Examination of Eigenvalues and the scree plot suggested four factors. We removed six items (items 7–11 and 23) in the scale due to cross-loadings and/or low communalities.

We conducted a second EFA with the remaining 17 items. The Eigenvalues associated with the first two factors (9.02 and 2.36) and screen plot suggest a two factor solution. The two factor solution accounted for 62.57% of the variance, and this combination of items (6 behavioral and 11 affective) did not demonstrate large cross-loadings. The six behavioral and 11 affective items loaded onto the respective factors (see Table 1). Strong relationships (i.e., ranging from 0.70 to 0.90) were observed between each item and its respective dimension, and a moderate, positive correlation was observed between the combined behavioral and affective dimensions of the measure (*r* = 0.53). We also found high internal consistency reliabilities for each dimension (*α* = 0.89 for the behavioral dimension, *α* = 0.95 for the affective dimension), as well as for the two combined scales (*α* = 0.94). These results lend support to our prediction that the construct has two underlying dimensions. In addition, we found high internal consistency (*α* > 0.80) for all other measures (see Table 2 ^3^).

**Table 2:** Correlations between study variables.

	SI-beh.	SI-affect.	SI-overall	Social conn.	Job sat.	Friendship	NA	PA.	Extra.	Happiness	Isolation	Social support
SI-beh.	(0.89)											
SI-affect.	0.53^**^	(0.95)										
SI-overall	0.79^**^	0.94^**^	(0.94)									
Social conn.	0.35^**^	0.61^**^	0.58^**^	(0.93)								
Job sat.	0.19^**^	0.57^**^	0.49^**^	0.48^**^	(0.92)							
Friendship	0.62^**^	0.78^**^	0.82^**^	0.68^**^	0.55^**^	(0.91)						
NA	0.02	−0.37^**^	−0.27^**^	−0.55^**^	−0.75^**^	−0.39^**^	(0.87)					
PA	0.24^**^	0.60^**^	0.53^**^	0.42^**^	0.80^**^	0.56^**^	−0.62^**^	(0.93)				
Extra.	0.23^**^	0.46^**^	0.43^**^	0.45^**^	0.34^**^	0.51^**^	−0.33^**^	0.38^**^	(0.88)			
Happiness	0.16^*^	0.37^**^	0.33^**^	0.43^**^	0.56^**^	0.43^**^	−0.52^**^	0.55^**^	0.37^**^	(0.81)		
Isolation	−0.47^**^	−0.69^**^	−0.69^**^	−0.57^**^	−0.47^**^	−0.81^**^	0.32^**^	−0.51^**^	−0.45^**^	−0.35^**^	(0.88)	
Social support	0.55^**^	0.64^**^	0.67^**^	0.54^**^	0.51^**^	0.72^**^	−0.39^**^	0.55^**^	0.43^**^	0.43^**^	−0.70^**^	(0.86)

*N, 188. SI-behav., behavioral dimension of workplace social interactions (SI) measure; SI-affect., affective dimension of workplace SI measure; SI–overall, combined behavioral and affective dimensions of workplace SI measure; social conn., social connectedness; Job satis., job satisfaction; NA, negative affect; PA, positive affect; extra, extraversion*.

* *p < 0.05*;

** *p < 0.01*.

### Convergent and Discriminant Validity

A correlation matrix consisting of the correlations between the newly developed scale and the related scales was created (see Table 2; Schmitt and Klimoski, 1991; Hinkin, 1998). The two dimensions of the proposed measure and the social connectedness are moderately and positively correlated (*r* = 0.59), indicating that they are tapping similar constructs. The affective dimension of the measure is more strongly related (*r* = 0.61) to the social connectedness scale than is the behavioral dimension (*r* = 0.35). We used Steiger’s *Z* test to determine the significance of this difference. Results showed that the affective dimension has a significantly stronger relationship with social connectedness than the behavioral dimension, suggesting that the behavioral dimension may be capturing a larger portion of the construct space that has not been previously measured. Also as expected, both of the dimensions of the proposed scale and the overall composite measure also showed a positive and moderate correlation with social support (*r* = 0.55 for behavioral; *r* = 0.64 for affective; *r* = 0.67 for combined).

Compared to measures of social connectedness and social support, the proposed measure was, in general, more highly correlated with the measure of workplace friendship (*r* = 0.62 for behavioral, *r* = 0.78 for affective, and *r* = 0.82 for combined). Notably, Steiger’s *Z* test indicated that the affective dimension has a significantly stronger correlation with workplace friendship compared to the behavioral dimension (*p* < 0.001).

As expected, both of the dimensions and the overall composite measure negatively correlated with workplace isolation (*r* = −0.47 for behavioral; *r* = −0.69 for affective; *r* = −0.69 for combined). Steiger’s *Z* test suggested that the affective dimension has a significantly stronger relationship with workplace isolation (*p* < 0.001), suggesting that the behavioral dimension is more distinct.

Finally, correlations indicated that the proposed measure is distinct from extraversion, with the pattern of correlations suggesting a moderate positive association between extraversion and both the behavioral and affective dimensions of the measure (*r* = 0.23 and *r* = 0.46). Steiger’s *Z* test indicated that the affective dimension has a significantly stronger correlation with extraversion compared to the behavioral dimension (*p* < 0.001).

We also tested discriminant validity using the method proposed by Fornell and Larcker (1981). We examined the square root of the average variance extracted (AVE) for each of our newly developed latent variables, and compared them to the bivariate correlations between our measures and all other similar constructs. The square roots of the AVE for the behavioral and affective dimensions were 0.76, and 0.78. These indices were greater than the bivariate correlations between this dimension and all other latent variables under examination, except for workplace friendship (*r* = 0.78). Thus, the behavioral measure demonstrated acceptable levels of discriminant validity, and the affective measure showed discriminant validity for most previously developed measures. Further examining the distinctiveness of the current measure from friendship, an EFA was conducted using the items from the newly developed measure and from the Workplace Friendship Scale. Results indicated that four factors account for 69.1% of the variance: the behavioral dimension of the new scale, the affective dimension of the new scale, and two dimensions of the existing Workplace Friendship Scale (Prevalence and Opportunities). All of the items from the friendship measure loaded onto separate factors, with the exception of one item. This item, “I enjoyed making friends with people in my organization,” loaded onto both the behavioral dimension (0.49) of the proposed measure and the friendship Prevalence dimension (0.38). As a result, this item was subsequently removed from the proposed measure. Taken together, these results provide support for the distinctiveness of the friendship measure compared to the newly developed scale.

### Confirmatory Factor Analyses

To further assess the dimensionality of the newly developed measure, two confirmatory factor analyses were conducted using the remainder of the sample (*N* = 315). In the first analysis, a two-factor model was fit to the data (*χ*^2 (118)^ = 367.859, *p* < 0.001). Global fit indices for the two-factor model were acceptable: CFI = 0.94, RMSEA = 0.08, SRMR = 0.04. In a second analysis, a one-factor model was fit to the data (*χ*^2 (119)^ = 1041.441, *p* < 0.001). Global fit indices for the one-factor model were not acceptable (Hu and Bentler, 1999): CFI = 0.78, RMSEA = 0.16, SRMR = 0.09. Given that the two-factor model exemplified acceptable overall fit, we examined the parameter estimates. Examination of the standardized factor loadings revealed that all items appropriately loaded well onto their respective dimension (i.e., behavioral or affective). All item loadings exceeded 0.74 and significantly differed from zero (*p* < 0.01), confirming that each of the two factors is well-defined by its items.

### Predictive Validity

We examined incremental predictive validity with the first sample (*N* = 188) using hierarchical regression analyses (Cronbach and Meehl, 1955; Hinkin, 1998). Specifically, we determined whether our newly developed measure predicted additional variance in these criterion measures (job-related affective well-being, happiness, job satisfaction) above and beyond the Lee and Robbins’ (1995) measure of social connectedness given its predominance in the literature (Cohen and Cohen, 1983; Hunsley and Meyer, 2003).

Controlling for teleworking and positional tenure, results show the newly developed measure significantly predicted job satisfaction (Δ*R*^2^ = 0.07, *β* = 0.32, *p* < 0.01) and positive job-related affect (Δ*R*^2^ = 0.12, *β* = 0.43, *p* < 0.01) above and beyond the measure of social connectedness.^4^ However, the proposed measure did not provide incremental validity over the social connectedness measure in predicting negative job-related affect (Δ*R*^2^ = 0.00, *β* = 0.08, *p* = 0.30) or happiness (Δ*R*^2^ = 0.01, *β* = 0.14, *p* = 0.10).

To determine whether the pattern of results was similar across the two samples, we examined the same intercorrelations and regression analyses described above using the other portion of the sample. The conclusions from those analyses were identical to those using the first portion of the sample with respect to convergent, discriminant, and predictive validity.^5^

## Discussion

The results provide initial evidence supporting the psychometric properties and predictive validity of a measure of informal, non-task-related workplace social interactions. Analyses supported our prediction that the items would exhibit a two-factor structure composed of affective and behavioral dimensions. The new measure has a sufficient number of items for each dimension (six for the behavioral dimension and 10 for the affective dimension) and demonstrates high internal reliability. The new measure shows evidence of appropriate convergent and discriminant validity with other constructs as expected (i.e., social connectedness, workplace friendship, workplace social support, extraversion, workplace social isolation). Additionally, the measure accounted for additional variance in job satisfaction and positive job-related affect above and beyond a commonly used, existing measure of social connectedness.

Findings concerning the distinctiveness of the behavioral and affective dimensions of the measure warrant further discussion. The behavioral dimension demonstrated relatively weaker relationships with the constructs we included as compared to the affective dimension. For instance, we found that the behavioral dimension has a positive but weak correlation with job satisfaction (*r* = 0.19). In contrast, the affective dimension exhibits a much stronger relationship (*r* = 0.57) with job satisfaction. This distinction may suggest that behavioral interactions are a necessary, but insufficient, means for increasing job satisfaction: one must also experience increases in positive affect associated with social interactions to achieve gains in job satisfaction. Indeed, although the behavioral items reflect the types of interactions normally thought of as conducive to positive affect, our study suggests that these favorable types of events may be weakly related to more distal outcomes like satisfaction and (arguably) job-related positive affect.

Regarding predictive validity, results show that the overall composite demonstrated incremental validity over social connectedness in predicting job satisfaction and positive job-related affective well-being, but not in predicting negative job-related affective well-being and happiness. Our measure may not have improved the prediction of happiness because happiness is a broader construct, whereas our measure is more narrowly focused on interactions and resultant emotional experiences. Indeed, previous scholars have underscored the importance of matching the specificity of predictors and criterion (e.g., Van Iddekinge and Ployhart, 2008), and it seems that the specificity of the newly developed measure may have reduced its ability to predict general levels of happiness.

### Limitations and Future Directions

Our results provide initial evidence for the two-factor structure of a newly developed measure of informal workplace social interactions. It is important to validate these results with additional samples; in particular, organizational samples. Moreover, the survey data were self-reported; therefore, it would be prudent to also evaluate data from secondary sources. Future research could also examine additional variables potentially impacted by workplace social interactions, as we included a limited range of predictors and outcomes to assess the predictive validity of our new measure. Finally, researchers may consider using other ways of assessing the occurrences of social interactions using more advanced technology (e.g., counting interactions, wearable sensors) to supplement or validate survey methods.

In conclusion, this study developed a measure of informal workplace social interactions consisting of two distinct behavioral and affective dimensions. We hope that this measure will serve as a psychometrically valid measure for future research on the relationships between workplace social interactions and individual and organizational outcomes.

## Data Availability

The data used in this study are available upon request to the corresponding author.

## Ethics Statement

The study was carried out in accordance with the recommendations of the Institutional Review Board of George Mason University which approved the study. All subjects gave informed consent in accordance with the Declaration of Helsinki.

## Author Contributions

All authors identified the need for this measure. CW analyzed the data and drafted most of the manuscript. IS collected and analyzed the data, and revised/edited the manuscript. AA revised/edited the manuscript. SK oversaw the project and the data collection/analysis. SM revised/edited the paper.

### Conflict of Interest Statement

The authors declare that the research was conducted in the absence of any commercial or financial relationships that could be construed as a potential conflict of interest.

## Footnotes

^1^Item numbering is according to Table 1.

^2^We intended to select 200 participants, but a human error oversight led to the selection of 188 instead.

^3^Scale scores were computed by calculating the mean of all of the items within a scale. All items were retained for the scale scores, except for one item in the Subjective Happiness Scale (“Some people are generally not very happy. Although they are not depressed, they never seem as happy as they might be. To what extent does this characterization describe you?”). The reliability of the scale with this item was very low (*α* = 0.34), but increased to an acceptable level (*α* = 0.81) after the item’s removal.

^4^We also controlled for participant age and gender and found that the same pattern of results held.

^5^Due to space limitations, we could not include the results in the manuscript. Please contact the corresponding author for the full results.
